# Cognitive Refined Augmentation for Video Anomaly Detection in Weak Supervision

**DOI:** 10.3390/s24010058

**Published:** 2023-12-21

**Authors:** Junyeop Lee, Hyunbon Koo, Seongjun Kim, Hanseok Ko

**Affiliations:** 1 School of Electrical Engineering, Korea University, Seoul 02841, Republic of Korea; junyeoplee94@gmail.com; 2 Korea Institute of Civil Engineering and Building Technology, Goyang-si 10223, Republic of Korea

**Keywords:** weakly supervised video anomaly detection, feature augmentation, multiple instance learning

## Abstract

Weakly supervised video anomaly detection is a methodology that assesses anomaly levels in individual frames based on labeled video data. Anomaly scores are computed by evaluating the deviation of distances derived from frames in an unbiased state. Weakly supervised video anomaly detection encounters the formidable challenge of false alarms, stemming from various sources, with a major contributor being the inadequate reflection of frame labels during the learning process. Multiple instance learning has been a pivotal solution to this issue in previous studies, necessitating the identification of discernible features between abnormal and normal segments. Simultaneously, it is imperative to identify shared biases within the feature space and cultivate a representative model. In this study, we introduce a novel multiple instance learning framework anchored on a memory unit, which augments features based on memory and effectively bridges the gap between normal and abnormal instances. This augmentation is facilitated through the integration of an multi-head attention feature augmentation module and loss function with a KL divergence and a Gaussian distribution estimation-based approach. The method identifies distinguishable features and secures the inter-instance distance, thus fortifying the distance metrics between abnormal and normal instances approximated by distribution. The contribution of this research involves proposing a novel framework based on MIL for performing WSVAD and presenting an efficient integration strategy during the augmentation process. Extensive experiments were conducted on benchmark datasets XD-Violence and UCF-Crime to substantiate the effectiveness of the proposed model.

## 1. Introduction

In the realm of video surveillance, a system’s capacity to apprehend unlearned anomalies is paramount. Also, abnormal behavior can manifest beyond familiar training data sets. In light of these observations, anomaly detection holds a central role in identifying outlying data points and commands a significant position in various areas [1,2,3,4,5,6,7]. Its applications extend to the detection of irregular behaviors and states through image inputs, or the isolation of anomalous regions within images. Through concerted efforts in the research domain, surveillance systems have undergone refinement across various elements. This includes the acquisition of robust data features, temporal information incorporation for enhanced classification, preservation of normal behavioral patterns, and the establishment of a classification framework via regeneration or trajectory analysis [8,9,10]. Furthermore, data refinement procedures encompass adjustments or augmentations that are assimilated during the learning process to ensure optimal performance. Particularly, anomaly detection in video inputs has garnered substantial attention in recent years and can be categorized into three distinct fields based on the training dataset and detection methodology.

The one class classification (OCC) method focuses on training solely on normal state datasets and subsequently evaluates scores for data points deviating significantly from the established norm. This approach is well-suited for datasets where the distinction between normal and abnormal states is discernible [11,12,13,14]. Extensive research endeavors have been dedicated to addressing challenges associated with this method. Nevertheless, its limitation lies in its exclusive acquaintance with the normal state distribution, rendering it less effective for intricate datasets with high inter-correlation. Additionally, the potential for overfitting arises due to data imbalances.

In contrast, unsupervised detection endeavors to identify abnormal frames without any foreknowledge about the training dataset. This method faces challenges in accurately predicting the distribution of an unlabelled dataset, especially within the domain of deep learning, which inherently exhibits data-driven characteristics [15,16,17].

The most widely researched field within this domain is weakly supervised video anomaly detection (WSVAD), characterized by its reliance on weak supervision. This methodology entails searching for anomalous frames within a video stream, guided by labels assigned to the entirety of the video [18,19,20,21].

Weakly supervised anomaly detection, featuring binary video labels, stands as a cornerstone in practical applications. Diverse methodologies have emerged to address this challenge, with multiple instance learning (MIL) research emerging as a prominent avenue of investigation. MIL involves segmenting a video sequence into snippets or clip units, subsequently training a clip-level anomaly detector [22,23]. This entails learning to decrease the anomaly score for snippets bearing normal labels, and conversely, augmenting the predicted anomaly score for snippets originating from abnormal videos.

Existing MIL anomaly detection studies have primarily centered on minimizing the gap between the normal datasets. This approach seeks to establish a discriminative plane by narrowing the distribution of the normal dataset acquired during the learning process. Additionally, it endeavors to establish an unbiased discriminator by estimating the distribution of a relatively consistent normal dataset, as opposed to an abnormal dataset.

Yet, given the persistence of abnormal behavior emanating from a normal state, clustering centered around normal behavior in instances where normal traits are intermittently observable might lead to erroneous classification as normal behavior. Distinguishing abnormal behavior from normal behavior can often prove challenging when assessed from an abnormal perspective, leading to a decline in performance. Recognizing the temporal continuity of behavior, there exist instances among anomalies that mirror the initiation of behavior and resemble the normal dataset. Similarly, within test data classified as normal, there are samples akin to learned abnormal behavior.

**Figure 1:**
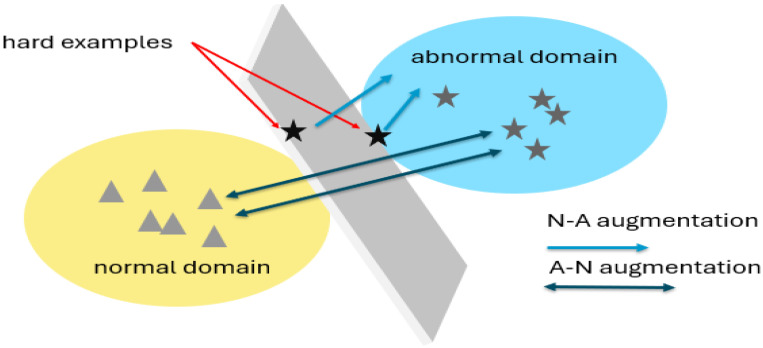
Illustration of the fundamental concept behind the two augmentations introduced in this study. The “hard examples” represent data points that lead to errors and diminish the overall accuracy. These challenging instances often reside at the interface between the normal domain and the anomalies domain, creating a classification hurdle along the discriminatory plane. To mitigate this issue, we propose two augmentations: N-A augmentation, which involves displacing anomalies towards the normal distribution, and A-N augmentation, which focuses on shifting data from the normal distribution toward the anomalies domain. These strategic augmentations are aimed at refining the classification process, particularly in handling “hard examples”.

In light of these considerations, we propose two augmentations, outlined in Figure 1, designed to bridge the gap between each domain. Through direct dataset augmentation, we aim to enhance learning performance. The N-A augmentation strategy transforms anomalies into reference points for normal snippets during the learning process, while the A-N augmentation approach increases the distance between the two domains based on abnormal snippets.

In pursuit of this objective, our focus lies in augmenting learning through distributions within both the normal and abnormal domains. The contribution of this work is summarized as follows:

- Introduces a novel augmentation technique designed to complement the memory MIL framework acquired through established loss functions. This augmentation process is geared towards unbiased learning, bolstering pertinent feature components within each domain.
- Proposes a multi-head feature attention (MHFA) module, demonstrating efficient integration of existing feature embeddings and augmented features. Each incorporated feature contributes significantly to performance robustness, presenting a discerning feature representation strategy within a MIL framework.
- Attains competitive performance on the extensive UCF-Crime [18] and XD-Violence datasets [24].

The structure of this study encompasses the following sections: abstract, introduction, related work, approaches, experiment, and discussion, each briefly explained as follows. In the abstract, an overview of the entire research content and concept is provided. The introduction outlines the necessity and background of the research, introducing the technologies employed in the research field. The related work section introduces studies related to the proposed sub-models in the research. Subsequently, the approaches and experiment sections present descriptions of the proposed research model and detail the experiments conducted. Finally, the discussion section provides the analysis of the study.

## 2. Related Work

Video anomaly detection has evolved in various forms, initially starting with the task of training on normal video clips to identify abnormal clips. In recent times, efforts have extended to identifying anomalies within video clips without labels. The weakly supervised video anomaly detection (VAD) proposed in this study is among the most practical and actively researched areas to date. In this approach, the network is trained only with video labels, lacking specific cues indicating abnormal behavior at particular locations. A recently proposed multiple instance learning method effectively addresses this challenge, providing a more efficient and robust solution compared to other approaches. Additionally, the field of feature extraction, actively explored for extracting temporal information throughout the task to detect abnormal cues in videos, plays a crucial role. Also, the memory module proposed in this paper for memory augmentation is particularly impactful in entire video anomaly detection fields, as it memorizes normal cues, distinguishing them from abnormal cues. The methods introduced in the following paragraphs serve as research areas that can play a vital role in weakly supervised VAD establishing logical connections and discussions for comprehensive exploration. In the section on related work, we delve into a comprehensive exploration of multiple technologies pertaining to the primary objective of our study, weakly supervised video anomaly detection. Initially, we introduce the types and methodologies of technologies employed in video anomaly detection (VAD). Subsequently, we present sub-model approaches as part of our endeavor to contribute valuable insights to the field.

### 2.1. Weakly Supervised Video Anomaly Detection

In video anomaly detection, three distinct approaches have emerged: one class classification (OCC), weakly supervised, and unsupervised methods. Among these, weakly supervised video anomaly detection (WSVAD) has undergone significant development through various techniques [25,26,27]. Early work by Sultani [18] introduced multiple instance learning for WSVAD, and subsequent endeavors incorporated graph convolution networks and clustering for detecting anomalous events. However, due to limitations in the distribution estimation capabilities of these models, recent studies have focused on obtaining unbiased spatial-temporal features. Novel methodologies involving transformers and I3D features have been proposed to address this challenge.

### 2.2. Multiple Instance Learning

Multiple instance learning (MIL) is a weakly supervised learning algorithm that selects crucial instances from training data grouped in instance form for learning. This approach proves effective when labels are provided not for individual instances but for the entire set of labels. MIL, including MIST and prior research, has demonstrated effective performance in video anomaly detection. In weakly supervised video anomaly detection, determining the precise time step of the occurrence of anomalies based on given video labels is needed. Therefore, constructing a MIL-based framework is reasonable, aiming to distinguish between normal and abnormal frames within videos containing abnormal labels. When utilizing MIL for anomaly video detection, the goal is to estimate the accurate temporal location, as video labels only determine the presence of an event. Initiatives such as Sultani’s research proposed a multiple instance ranking objective function to differentiate positive and negative bags in the early stages, progressing to the use of feature magnitude-based discrimination in RTFM. The proposed study introduces a baseline approach based on RTFM (Robust Feature Magnitude) learning, distinguishing abnormal instances by considering the top-k instances with high feature magnitudes. This approach enhances the probability of selecting abnormal instances in videos, subsequently improving training convergence. The outlined methodology highlights key distinctions from existing MIL (Multiple Instance Learning) studies employing classification score approaches, emphasizing the unique contributions of RTFM in addressing anomaly detection challenges.

### 2.3. Feature-Extration

Within the multiple instance learning (MIL) framework, I3D features extracted from videos are utilized, alongside the advance of various models to leverage features with spatial-temporal information such as LSTM, transformer, and Autoencoder. Notably, RTFM (2021) introduced feature magnitude learning, an effective approach for learning unbiased snippets. This involves training a classifier to identify the top-k snippets based on the feature magnitude of abnormal and normal data. In this study, inspired by the long-range capture capabilities derived from natural language processing (NLP) [28,29,30,31], we adopt a widely used transformer-based method to acquire local-global features. Additionally, we employ feature magnitude learning as a form of mild supervision to distinguish snippets effectively.

### 2.4. Memory-Module

The memory module plays a role in recording data patterns, thereby aiding in the classification of individual items. In the context of anomaly detection, where the identification of abnormal data is crucial, the ability to memorize the binary characteristics of data has been addressed through the implementation of a memory module in recent studies. The memory module utilized in this study aimed to distinguish between normal and abnormal instances. In prior research, the memory module involved embedding instances after feature extraction, training with renewed embedding vectors, and conducting classification during the inference phase by assessing similarity with input. As an illustration of leveraging multi-view features, HF2-VAD employed a memory auto-encoder that retained optical flow features to perform future frame reconstruction. Similarly, approaches such as AMMC-NET utilized prior knowledge of appearance and motion. However, employing various sub-features necessitates abundant multi-stage processing, posing a disadvantage. In this research, we adopt the i3d feature, extracting image and optical flow in combination rather than directly extracting optical flow and addressing the drawbacks of employing various sub-features.

### 2.5. Feature Augmentation

Data augmentation is commonly proposed as a solution for addressing data imbalance. In the realm of video anomaly detection, there exists a disparity in the number of instances representing features in normal and abnormal datasets, necessitating a selective refinement of features. Consequently, the role of a loss function guiding feature embedding in the augmentation aspect becomes crucial. For instance, HSC attempted a contrastive learning approach to refine the embedding in the memory bank. The study conducted augmentation through experimental combinations of various loss functions.

## 3. Approaches

**Figure 2:**
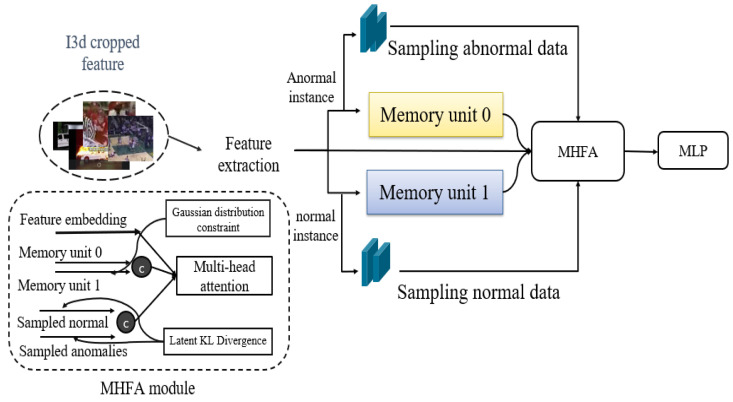
Overview of the proposed model. Initially, I3D cropped features undergo feature extraction using a multi-head attention structure. Subsequently, based on weakly supervised labels, Each of instances is further processed through a sampling unit and a memory unit, yielding inputs for the MHFA module. Features are integrated into an MLP unit for anomality score.

In this section, we propose an effective method for utilizing a memory unit in the multiple instance learning (MIL) framework. The research aims to enhance discriminative performance by leveraging information extracted not only from the conventional feature embedding but also from the memory unit, along with sampled normal and abnormal clips using the introduced memory-highlighted feature aggregation (MHFA) module. This approach is trained using the BCE loss, latent loss, and KL loss, which constitute the model to be introduced. Therefore, in this study, we present an augmentation process suitable for the memory MIL framework, anticipating performance improvement through the design of corresponding loss functions. The MHFA module is proposed to demonstrate efficient integration in the augmentation process. This approach can be applied to other modules within the existing framework, with the expectation of enhancing overall performance. In an overview of Figure 2, the proposed model has a structure that produces output through four sub-modules. The I3D-RGB cropped feature is used as input, the anomaly score is finally calculated as the output, and extraction is performed by passing the I3D-RGB cropped feature through a transformer structure. In addition, it is divided into normal instances and abnormal instances using video labels, passes through each sampled unit and memory unit, and merges them with existing features into a multi-head attention structure in the MHFA module to produce the final score.

### 3.1. N-A Augmentation and A-N Augmentation

Focusing on the attention of transformers, which acquire superior ability in capturing long-range dependencies and correlations in natural language processing (NLP), multi-head attention was used in the study to extract initial features and concatenate intermediate features [32,33].

(1) $$\begin{array}{c} X_{f}=softmax\left(\frac{QK^{T}}{\sqrt{D}}V_{i3d}\right) \end{array}$$

(2) $$\begin{array}{c} V_{f}=X_{f}*V_{i3d} \end{array}$$

(3) $$\begin{array}{c} V_{m}=M_{0}⊕M_{1} \end{array}$$

(4) $$\begin{array}{c} V_{s}=S_{a}⊕S_{n} \end{array}$$

Equations (1)–(4) present the formula for obtaining features, wherein $V_{f}$ is obtained through self-attention of $V_{i3d}$. $M_{0}$ and $M_{1}$ are the abnormal instance and normal instance embedded in the memory unit, respectively, while $S_{a}$ and $S_{n}$ are the samples that increase the distance between the abnormal instance and the normal instance, respectively. The formulation of $X_{f}$ is based on a transformer to induce self-attention in $V_{i3d}$, where *Q*, *K*, *D* represents the query, key, dimension each of $V_{i3d}$. Through Equations (1) and (2), we acquire refined VI3D features, and via Equations (3) and (4), we can integrate the sampled instances.

### 3.2. Sampling and Memory Unit

The details of the role and structure of the sampling unit and memory unit are described here. The sampling unit is two Conv layers learned with KL divergence loss and plays the role of sampling optimal distant features according to the learning process in the instance [34,35]. The memory unit embeds features by passing the features through the sigmoid function in the existing memory unit [14,36].

(5) $$\begin{array}{cc} \; & Y_{0}^{K}=Sigmoid\left(M_{0}^{k}*{V_{f}^{k}}^{T}\right) \end{array}$$

(6) $$\begin{array}{cc} \; & M_{0}^{\hat{k}}=Y_{0}^{K}*V_{f} \end{array}$$

Equations (5) and (6) are the memory update process, and *k* is the index of memory. Similar indices are obtained using top-k feature magnitude. The anomaly score is determined by the sigmoid of the product of $V_{f}^{k}$ and $M_{0}^{k}$, and is learned through cross-entropy loss. All instance inputs of normal labeled video are embedded into memory unit1. During the learning process, all data that have already been labeled as normal are normal frames, and the focus is on the normal/abnormal classification of anomaly labeled snippets.By utilizing the binary cross-entropy (BCE) loss function, effective embedding of instances in a normal state can be achieved. Consequently, this demonstrates the process of embedding instances fitting the memory through self-supervised labeling. The mapping of the refined $V_{f}^{k}$ at index *k* to indicate proximity to a normal instance, ranging between 0 and 1, is obtained through the sigmoid function.

### 3.3. MHFA Module

MHFA, the last model before calculating the final score, is a module to effectively concatenate the values obtained from the sub-module above. The meanings of each value are first, a memory feature that learns labels for each instance according to the normal situation estimated by Gaussian distribution in the memory unit, second, a sampled instance that maximizes the distance between normal and abnormal instances, and last, the original feature. The goal is to create rich yet discriminative features. Through this module, features with enhanced classification performance are obtained through attention to features concatenated for each index.

### 3.4. Total Loss

The total loss is calculated as the sum of the losses designed for each module as follows. The entire loss consists of the corresponding term that constitutes the MHFA module in Figure 2. These are BCE loss corresponding to feature embedding, latent loss corresponding to the memory unit, and KL loss corresponding to the sampling unit. The factors $\lambda_{1}$ and $\lambda_{2}$, experimentally set to 0.1 and 0.0005, respectively, play a role in aligning the scales of the binary cross-entropy (BCE) loss, latent loss, and Kullback–Leibler (KL) divergence loss. This adjustment is aimed at achieving balanced convergence during the training process.

(7) $$\begin{array}{cc} & total_{loss}=\lambda_{0}BCE_{loss}+\lambda_{1}Latent_{loss}+\lambda_{2}KL_{divergence} \end{array}$$

An explanation of each loss is provided in the following subsections.

#### 3.4.1. BCE Loss

Binary cross-entropy (BCE) loss is a loss for training memory unit0 and unit1, and each instance is embedded according to its weak label.

(8) $$\begin{array}{cc} & BCE_{loss}=BCE_{1}\left(Y_{n}^{0},y^{a}\right)+BCE\left(Y_{a}^{0},y^{n}\right)+BCE_{3}\left(Y_{n}^{1},y^{n}\right)+BCE_{4}\left(Y_{a}^{1},y^{a}\right) \end{array}$$

Looking at each term, the first and second terms mean the sigmoid score from memory unit 0 corresponds to the abnormal and normal labels, and the third and fourth terms mean the sigmoid score from memory unit 1. Although all normal inputs are classified as normal embeddings, the sigmoid score is used to train the source data of the normal label to a normal situation.

#### 3.4.2. Latent Loss

Latent loss is a KL latent loss that trains the latent space of normal data according to the Gaussian distribution and finds out-of-distribution (OOD) data. Because of this, constraint loss can play a role in assuming the distribution of normal situations and estimating abnormal data (OOD). We adopted the Gaussian distribution constraint, which is usually used in unsupervised situations, to observe the abnormal dataset from a normal perspective.

(9) $$\begin{array}{cc} & Latent_{loss}=KL\left(N\left(z^{n}|\mu^{n},\sigma^{n}\right)\|N\left(0,1\right)\right) \end{array}$$

The latent loss indicates distance between mean and variance of $z^{n}$, the $\mu^{n}$, and $\sigma^{n}$ from a Gaussian distribution N(0,1).

#### 3.4.3. KL Loss

Since KL divergence is a non-symmetric metric, relative entropy was measured using abnormal situations as the denominator, and information divergence was given a negative rating because it should increase as learning progresses. Additionally, the threshold must be set so that the value does not diverge infinitely. What we experimentally observed was that a threshold value that was too large actually hindered learning performance. This was observed as the sampled instance affecting other losses and acting as noise in the learning process. In other words, a certain amount of sampled instances are necessary, but if the sampled instances are far enough to interfere with other learning, it is confirmed that they serve as abundant features in the learning process.

(10) $$\begin{array}{cc} \; & kl-divergence=KL(q(z|x,y)\|p(z|y)) \end{array}$$

Inspired by HF2-VAD (Zhian Liu, 2021) [37], which increased the performance of frame reconstruction by reconstructing optical flow, latent loss and KL loss were proposed similar to ELBO used in conditional variational autoencoder (CVAE), which is similar to *z* in the posterior distribution. This is equivalent to sampling and approximating *p* and *q* with a Gaussian distribution. However, when *z* is called abnormal sampling, it cannot be approximated with a Gaussian distribution depending on the characteristics of OOD data, so we proposed an equation by simply increasing the distance between the two terms.

(11) $$\begin{array}{cc} & KL\left(P_{a}\|P_{n}\right)=-\sum_{\sigma }^{}P_{a}\left(\sigma \right)ln\frac{Pa(\sigma )}{Pn(\sigma )} \end{array}$$

(12) $$\begin{array}{cc} \; & KL_{loss}=max\left(0,m_{th}-KL\left(P_{a}\|P_{n}\right)\right) \end{array}$$

Therefore, $KL_{loss}$ is derived using Equation (10). $P_{a}$ is a measure of sampled abnormal data, and $P_{n}$ is a measure of sampled normal data. We also suggest threshold clipping to avoid diverging with minus infinity. In the experiment, we set the threshold to 1000 to ensure scale balance with other losses and experimentally optimal performance.

## 4. Experiment

### 4.1. Datasets

An experiment was performed using the UCF-Crime (Sultani et al. 2018 [18]) dataset and the XD-Violence (Wu et al. 2020 [24]) dataset, which are dominantly used in other anomaly detection work.

#### 4.1.1. UCF-Crime Dataset

UCF-Crime is an extensive 1900 surveillance video dataset containing 128 h of video and is a dataset that can be applied to public safety such as abuse, arrest, and arson. Unlike the Avenue and Shanghai Tech datasets, which have street views, it is a practical dataset due to that fact that the abnormal behavior that occurs in the view is of a certain size.

#### 4.1.2. XD-Violence Dataset

XD-Violence dataset (Wu et al. 2020 [24]) is a multi-modal modality set with a duration of 217 h and 4754 video and audio sets. This is video collected from movies, sports, games, hand-held cameras, CCTV, etc. It has nice violence types and has robust characteristics, but the size of the people is not constant.

#### 4.1.3. Evaluation Metric

Following the evaluation metric widely adopted in the field of video anomaly detection, we applied and measured the ROC curve (AUC) to the UCF-Crime dataset. On the other hand, in the XD-Violence dataset, we measured the widely used precision recall (AP) and compared it with other SOTA models.

#### 4.1.4. Implemenation Details

We used the I3D snippet feature pretrained with Kinetics-400, and as experimental hyperparameters, we used a memory unit with a feature length of 1024, a batch size of 128, and a dim of 512. Also, λ0, λ1, λ2 of 1, 0.1 and 0.0005, respectively, were used. The size of λ was determined in each experiment by considering the magnitude of the loss when convergence was achieved. Although experiments were conducted with various values of λ, it was found that the proposed total loss configuration, considering the linear combination of the loss functions, appears to be reasonable, incorporating a balance factor. The relationship among the three variables adjusts the rate of decrease during the optimization process. The experiments were conducted in a PyTorch environment, utilizing a NVIDIA Geforce Single RTX 3090 GPU (NVIDIA, Santa Clara, CA, USA), 64 GB RAM, and an Intel Core i9 CPU (Intel, Santa Clara, CA, USA).

### 4.2. Quantitative Result

Table 1 shows the AUC performance for UCF-Crime and shows that the proposed method increased the baseline RTFM by 0.42% and achieved competitive results among the SOTA models.

Table 2 is a comparison between AP performance models for the XD-Violence dataset, and since XD-Violence is a multi-modal dataset, there is a feature that reflects the audio results. Like UCF-Crime, competitive frame-level AP performance was obtained, and the difference from RTFM is achieved with a difference of 1.71%. The best performance is indicated in bold.

### 4.3. Ablation Studies

Table 3 and Table 4 are the AUC measurement tables when varying the parameters of the abs term and sampling dim of KL divergence in the UCF-Crime dataset. The highest performance was measured in Experiment #6 and is indicated in bold. The performance was found to be optimal when the sampling dim was 256 and the abs term 500.

When comparing Experiment #1 with other experiments, there was an augmentation effect when applying KL divergence loss after sampling, and the change in abs term had a greater impact on optimal performance than the change in sampling dim.The objectives of Table 3 and Table 4 are to investigate the impact of the sampling dimension and absolute term parameter on experimental performance, determine their significance as parameters, and identify the optimal parameters leading to the best performance. According to the results in the tables, the absolute term effectively conducts sampling of distant normal and abnormal instances, while the sampling dimension indicates which dimensions are involved in the process. Table 5 presents data measuring the memory consumption of each module in the entire network. The proposed approach in this study utilizes a memory module for the MIL framework, necessitating the measurement of the computational load imposed by the proposed memory module across all modules. To calculate the overhead, the number of parameters for each module was measured. The parameters computed due to the memory module were found to be approximately 4.5% of the total module parameters, indicating a minimal computational load. Furthermore, the total parameters of the entire module amount to approximately 5.3 million, classifying it as a compact model. It was observed that the feature extraction and preprocessing components contribute the most significant memory consumption within the model.

## 5. Discussion

This study presents an experimental validation of the proposed video anomaly detection framework using extensive datasets from UCF-Crime and XD-Violence. The incorporation of sampling, memory data augmentation, and MHFA block demonstrates a direct and positive impact on the performance metrics.

Through ablation studies, it was established that fine-tuning hyperparameters significantly influences experimental outcomes, underscoring the need for tailored adjustments of sampling dimensions and thresholds for each dataset.

Augmentation strategies that facilitate the learning of distinct features were identified as pivotal in optimizing performance within the domain of weakly supervised video anomaly detection. This augmentation not only enriches memory unit embedding but also maintains critical distinctions from both normal and abnormal perspectives.

Furthermore, the study emphasizes the crucial role of dataset distribution in achieving clear classification performance in WSVAD. This inherently data-driven characteristic prompted the exploration of robust classification performance, which was substantiated through various experiments.

Despite the data-driven nature, the proposed method achieved features with rich and clear classification performance, demonstrating competitive results through straightforward yet effective data augmentation techniques. The applicability of this method extends to other state-of-the-art models, with potential for further performance enhancements.

## 6. Conclusions

This study has successfully demonstrated the efficacy of integrating data augmentation and augmented data to enhance feature-level classification performance. Leveraging KL divergence, we extracted characteristic features that significantly extended the domain separation. Furthermore, we employed Gaussian distribution estimation to augment the memory embedding unit, facilitating the effective discrimination between abnormal and normal instances. This proposed methodology not only enriched the feature set but also offered a versatile technique that can readily be implemented in diverse models. The extensive experimental evaluation of the UCF-Crime and XD-Violence datasets yielded competitive results, as evidenced by the achieved AUC and AP scores. The research results confirmed our novel contribution to the advancement of anomaly detection techniques in video analysis.

## Figures and Tables

**Table 1:** Frame-level AUC performance for UCF-Crime. Depending on the approach, we divided the table into unsupervised and weakly-supervised methods, delineating the AUC performance on the overall test set. The best results are displayed in bold.

Approaches	Method	Feature (RGB)	AUC (%)
Unsupervised	Hasan et al. [38]		50.60%
	kim et al. [16]	ResNext	52.00%
	BODS		68.26%
	GODS		70.46%
	GCL	ResNext	71.04%
WSVAD	Sultani et al. [18]	C3D	75.41%
	Zhang et al. [19]	C3D	78.66%
	Noise Cleaner	C3D	78.27%
	GCN	C3D	81.08%
	Wu et al. [24]	I3D	82.44%
	DAM	I3D	82.67%
	CLAWS	C3D	83.03%
		ResNext	82.61%
	RTFM	I3D	84.30%
	**Ours**	**I3D**	**84.72%**

**Table 2:** Frame-level AP performance for XD-Violence. The best results are displayed in bold.

Approaches	Method	Feature (RGB)	AP (%)
WSVAD	Wu et al. [24]	RGB	67.19%
		RGB	73.20%
	Sultani et al. [18]	RGB	75.53%
	HL-Net	RGB	73.67%
		RGB + Audio	78.64%
	CRFD	RGB	75.90%
	RTFM	RGB	77.81%
	MSL	RGB	78.28%
	MSL	RGB- VideoSwin	79.19%
	**Ours**	**RGB-I3D**	**79.52%**

**Table 3:** Frame-level AP performance for UCF-Crime w.r.t abs term. The best results are displayed in bold.

on UCF-Crime				
**abs Term**	**KL Loss**	**Sampling Dim**	**AUC**	**Experiment Number #**
	x		0.822	#1
1000	o	512	0.833	#2
500	o	512	0.8442	**#3**
300	o	512	0.8316	#4

**Table 4:** Frame-level AP performance for UCF-Crime w.r.t sampling dim. The best results are displayed in bold.

on UCF-Crime				
**Sampling Dim**	**KL Loss**	**abs Term**	**AUC**	**Experiment Number #**
128	o	500	0.8386	#5
256	o	500	0.8472	**#6**
512	o	500	0.8442	#7
1024	o	500	0.833	#8

**Table 5:** Parameter measurement per modules.

Number of Parameters			
**Network**	**Input Channel**	**Param**	**Percentage %**
Feature extraction	1024	1,573,376	27.2
Transformer module	512	1,048,576	18.1
Memory module	512	262,656	4.5
Entire network	1024	5,774,848	100

## Data Availability

The corresponding training and testing video data has been acquired through https://webpages.charlotte.edu/cchen62 and https://roc-ng.github.io/XD-Violence/ (accessed on 7 October 2023).
